# Supplementary material for: MetaRibo-Seq measures translation in microbiomes
Source: Nat Commun. 2020 Jun 29;11:3268. doi: 10.1038/s41467-020-17081-z (PMC7324362; doi:10.1038/s41467-020-17081-z)
Supplement: Supplementary file 10 — Supplementary Data 7 [file 41467_2020_17081_MOESM10_ESM.zip › File2/Confidence_High_Taxonomy/156306_out.krona.html]

Javascript must be enabled to view this page.

members
magnitude
magnitudeUnassigned
count
unassigned
taxon
rank

156306\_out

4

2
4
superkingdom

phylum
976
4

class
200643
4

4
171549
order

family
171552
4

838
4
genus


SRS012849\_contig\_number\_4035
2292054
1
species

species

SRS012969\_contig\_number\_31000SRS023526\_contig\_number\_32581SRS077849\_contig\_number\_12986
3
2292365
